# Navigating water crisis, cholera, and refugee context: Lessons from Nakivale refugee settlement, Isingiro District, Uganda

**DOI:** 10.1371/journal.pgph.0004201

**Published:** 2025-02-04

**Authors:** Freda Loy Aceng, Bonny Kintu, James Habumugisha, Stephen Kayanja, Flavia Wanyoto, Moureen Asiimire, Emmanuel Maurice Ochien, Bernard Amaga, Sam Kwesiga, Sylivia Kemigisha, Stella Maris Lunkuse, Barbara Namara, Edison Tumusherure, Allan Muruta, Godfrey Bwire

**Affiliations:** 1 Department of Integrated Epidemiology, Surveillance and Public Health Emergencies, Ministry of Health, Kampala district, Uganda; 2 Baylor College of Medicine Children’s Foundation, Kampala district, Uganda; 3 Nakivale Refugee Settlement, Isingiro district, Uganda; 4 Department of Environmental Health, Ministry of Health, Kampala district, Uganda; 5 World Health Organization, Kampala district, Uganda; 6 Isingiro District Local Government, Isingiro district, Uganda; 7 Nsamizi Training Institute of Social Development, Mpigi district, Uganda; University of Oslo Faculty of Medicine: Universitetet i Oslo Det medisinske fakultet, NORWAY

## Abstract

Inadequate safe water supply and cholera outbreaks are significant contributors to ill health in displaced populations, exacerbating the vulnerability of refugees fleeing conflicts. In November 2021, the Nakivale Refugee Settlement faced a cholera outbreak and water scarcity, putting displaced persons at risk of ill health. This study aimed to investigate the lessons learned from this incident and identify pathways to prevent future outbreaks, ultimately safeguarding the lives and well-being of vulnerable populations. A descriptive cross-sectional study analysed epidemiological data from the Uganda Ministry of Health, investigating the cholera outbreak among refugees in Nakivale Refugee Settlement, Isingiro district, from November 3rd to December 2nd, 2021. The outbreak affected 173 refugees, primarily in Nyarugugu B and C villages, with consumption of contaminated lake water identified as the likely cause. Inadequate access to clean water and sanitation facilities exacerbated the outbreak, which disproportionately affected individuals aged 10-19 years (30.1%) and females (65%). Promotion of safe water, sanitation, and oral cholera vaccination campaigns effectively controlled the outbreak. This study highlights the critical importance of providing refugees with access to safe water and sanitation infrastructure to prevent cholera outbreaks. Timely implementation of oral cholera vaccination campaigns and enhanced water management systems are crucial to preventing future outbreaks in refugee settlements.

## Introduction

In the intricate web of global health challenges, the delicate balance between water availability and disease outbreaks looms large [1,2]. Cholera, driven by the bacterium *Vibrio cholerae*, thrives where clean water remains elusive and sanitation systems falter [3,4]. Its repercussions ripple through vulnerable communities, amplifying existing hardships. Globally it has been estimated that each year there are about 1.3 to 4.0 million cases, and 21,000 to 143,000 deaths due to cholera [5]. According to the World Health Organisation (WHO), the risk of cholera at global level was assessed as very high in 2022, with over 29 countries reporting cases or outbreaks particularly in the WHO Regions of Africa (WHO-AFRO) and the Eastern Mediterranean [6]. Between January 1, 2022, and July 23, 2023, the WHO-AFRO reported 217,186 cholera cases and 4,002 related deaths, resulting in a case fatality ratio (CFR) of 1.8% [5].

Cholera is an acute diarrhoeal infection caused by consumption of food or water contaminated with the bacterium *Vibrio cholerae* [7]. The most effective strategies to control cholera include; improved access to safe drinking water and sanitation infrastructure, improved access to proper and timely case management of cases, improved infection prevention and control in healthcare facilities as well as promotion of preventive hygiene practices and food safety in affected communities [6]. The oral cholera vaccination (OCV) should be used concurrently with improvements in water and sanitation to control cholera outbreaks and for prevention in targeted areas at high risk for cholera as well as endemic areas [6,7]. WHO recommends that Member States reinforce and sustain cholera surveillance particularly at the community level, so as to detect suspected cases early, provide suitable treatment and prevent its spread. Early detection and treatment can reduce cholera’s case fatality ratio (CFR) to less than 1% [6].

Cholera outbreaks in Africa have been fuelled by inadequate Water Sanitation and Hygiene (WASH), natural disasters, conflict, and concurrent disease outbreaks [5]. Among the cholera-vulnerable populations, refugees significantly contribute to the reported cases and deaths [8]. Displaced from their homes, they often find themselves caught in the crossfire of both water scarcity and cholera outbreaks. Cholera outbreaks have occurred in refugee settlements in Uganda in previous years linked to poor water and sanitation status [9,10]. Here we explore the lessons drawn from this confluence, seeking pathways to prevent future similar outbreaks and safeguard lives and well-being of persons in such settings with vulnerable persons.

## Methods

### Study design

This was a descriptive study of the cholera outbreak that occurred in Nakivale Refugee Settlement.

### Outbreak location

Nakivale Refugee Settlement is located in Isingiro district in South West Uganda and the population in 2023 was 171,387. It was established in 1958 and officially recognised as a refugee settlement [11,12]. Isingiro district has 15 sub counties with Nakivale settlement spreading across Ngarama, Kashumba, Rugaaga and Rushasha sub counties. The settlement mostly spreads out to the North in order to access the water resources of Lake Nakivale [13].

At Nakivale refugee settlement, there is an individual in charge of health and political supervision under the office of the Prime Minister (OPM). The main organisation that is implementing health and surveillance is Medical Teams International headed by a medical coordinator. All health facility managers report to the medical coordinator who together with the OPM coordinator report to the District Health Office. There are eight health centres at the settlement including Rubondo health centre HC II, Juru HC II, Kibengo HC II, Rulongo HC II, Ruhoko HC III, Kabazana HC III, Nshungyezi HC III and Nakivale HC III. During cholera outbreaks, Nakivale HC III has an improvised tent which constitutes a treatment centre.

The disease surveillance reporting system follows a tiered approach from the community to the national level through the district health information system Version-2 (DHIS2) which is a web-based open source health management information system for gathering, validation, analysis and presentation of aggregate and patient-based statistical data [14]. Surveillance is also done by village health teams who conduct door-to-door surveillance through home visits and trainings are conducted by health workers. All alerts are reported to health assistants for verification using the case definitions under the Integrated Disease Surveillance and Response. If the case meets the case definition, then the procedure of reporting is followed. There is a facility surveillance committee that reviews disease incidences and a rapid response team that responds to outbreaks.

### Study variable

The variables considered in this study included; the demographics, date of onset, date of admission, date of discharge, outcome, signs and symptoms of cholera.

### Case definitions and case finding

A suspected case was defined as acute onset of watery stool, vomiting, abdominal pain, dehydration in a resident of Nakivale Refugee Settlement (or one with history of travel to Nakivale Refugee Settlement) from 4th November 2021 onwards. A confirmed case was a suspected case with laboratory confirmation of *Vibrio cholerae* from a stool sample by culture. We reviewed medical records and conducted active community search in the affected villages to map the case-patients’ locations and identify additional cases. The variables captured in the line list included; the patient demographics, dates of onset, admission and discharge, signs and symptoms, clinically or laboratory confirmed and outcome.

### Water sources or types

The water sources selected include motorised boreholes and surface water. These were randomly selected from the affected villages where cholera suspects were identified.

### Water collection methods

The community collects water using various kinds of containers such as jerrycans, buckets from the water sources.

### Water testing

In response to the outbreak, bacteriological water testing was done on the water sources used by the Nyarugugu community. A total of 8 water samples were collected including 4 from the household of suspected cholera cases, 2 from boreholes and 1 from the lake. These were all tested for water quality to identify microbial/faecocoliform contamination (*Escherichia coli*) alongside distilled water as a standard. Sterile bottles were used to collect water samples and for lake water a sampling cup was dipped inside the lake. The volume of the samples collected was 500 ml. The sampling point in lake Nakivale was at fetching points of villages with cholera cases. Random selection of the sampling point was done.

### Laboratory analysis

#### Faecal coliform determination using membrane filtration method using lauryl sulphate broth.

Water samples were collected and stored in a refrigerator overnight before analysis. This was done to avoid any alternations in the bacteriological status. A water sample of 100ml or its equivalent was filtered through a membrane filter composed of cellulose esters which retains all the bacteria on the surface of the membrane, which is then incubated with the girded side upon a selective medium.

#### Requirements.

Lauryl sulphate broth. 38.1 g of anhydrous broth powder is dissolved in 500ml of water. The solution is sterilized in an autoclave for 15 min at 121°C 15 bars. Remove media and store in a dry place.

Filtering unit, membrane filter pads, autoclave, incubator, Petri dishes, forceps, pipettes (graduated), transfer pipette, digital counter, water still, measuring cylinders, sterilizing burner, hot plate, disinfectant (ethanol 70%), weighing balance, thermometer.

#### Procedure.

The membrane filter is picked using sterile forceps ascetically into the Petri dish. Two millilitres of lauryl sulphate broth is transferred on to the absorbent pad in the Petri dish so that it is soaked just to leave a film of broth round the absorbent pad. Avoid over flooding the Petri dish with the broth. The membrane filter is put in the filtration unit with the gridded face up and replace the filter jar. One hundred millilitres or an equivalent of sample is poured into the filtration jar and all is filtered through the filter paper. The filtering membrane is removed and placed on to the absorbent pad that was earlier socked with the broth. The Petri dish is covered with the lid upper most, marked and placed it on to the Petri dish carrier. All the above is repeated for each sample and the Petri dish carrier is put in an incubator at 35^o^C. After incubation remove the carrier plus the Petri dish and allow it to cool for 10 minutes, to allow false yellow colour to lose colour and ensure that the yellow is only for the typical colonies. A magnifying glass and a counting pen may be used to count the colonies. Count all the yellow, pale yellow colonies which are convex, dome shape colonies with a reflecting surface as true colonies. These colonies are counted and reported as counts/100 ml of faecal coliforms.

#### Environmental assessments.

We conducted an environmental assessment of water sources including latrine coverage around the affected villages of Nyarugugu A, B and C. This was done through observations during the field visits, discussions with key informants, community members and no standardised data collection tool was used.

#### Data management and analysis.

The data was entered into Excel spreadsheets, cleaned and exported to Epi Info version 7.2.5.0 (US Centers for Disease Control and Prevention) for analysis, S1 Data. Descriptive findings were presented as frequency distributions, percentages and rates. Maps were drawn using QGIS (Quantum Geographic Information System) software.

#### Ethical consideration.

This investigation was in response to a public health emergency and was therefore determined to be non-research. The MoH through the office of the Director General of Health Services gave the directive and approval to investigate this outbreak. We used unique identifiers to ensure confidentiality.

## Results

### Detection and reporting of the outbreak

On 4^th^ November 2021, Uganda Ministry of Health (MOH) was notified of 15 suspected cholera cases from Nakivale Refugee Settlement in Isingiro District. From 4th – 11th November, at least 125 people had been affected, seven tested positive on Rapid Diagnostic Test (RDT) and there were no fatalities. A team from the Ministry of Health and Isingiro District Health team set out to determine the scope of the outbreak, identify possible exposures, recommend evidence-based control measures and perform reactive oral cholera vaccination campaign.

### The index case

The index case was a 35-year-old female from Nyarugugu C village admitted in Nakivale HC III on 4th November 2021 with a history of sudden onset of multiple episodes of loose watery motions with associated severe abdominal pain and multiple episodes of non-projectile watery vomitus. All the other history was unremarkable. At the time of admission, she had cold extremities with a temperature of 35.6°C, pulse of 140bpm and blood pressure of 75/40mmhg. A diagnosis of suspected cholera was made, and treatment initiated after sample collection. Two more patients from the same household of the index case also developed similar symptoms in the morning and were taken to the health centre. Several other households had individuals with similar symptoms.

### Descriptive epidemiology

#### Place characteristics.

**Fig 1:**
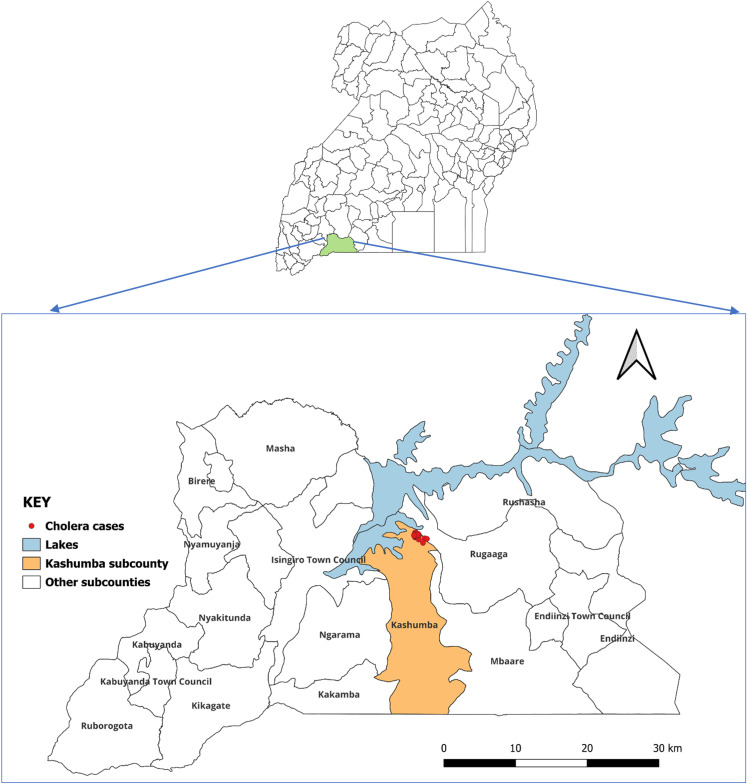
Map of Uganda showing Isingiro district. Base map shapefile obtained from Uganda Districts Shape files 2020, https://data.unhcr.org/en/documents/details/83043.

The cholera outbreak occurred in Nakivale Refugee Settlement. The cumulative cases including suspected and confirmed cases were 173. All the cases were from eight villages in Kashojwa parish in Kashumba sub county, Isingiro district (Fig 1). The affected villages included, Nyarugugu A, Nyarugugu B, Nyarugugu C, Base Camp, Kabazana A, Kashojwa B, New Congo and Sangano. The overall attack rate was 37 cases/10,000 population. The attack rates were highest in Nyarugugu B (180 cases/10,000 population) and Nyarugugu C (92 cases/10,000 population) (Table 1).

**Table 1:** The affected villages in Kashojwa parish, Kashumba sub county.

Village	Frequency	Percent	Population	Attack Rate/10,000
Nyarugugu B	67	38.73%	3,720	180
Nyarugugu C	96	55.49%	10,440	92
New congo	2	1.16%	1,040	19
Nyarugugu A	1	0.58%	1,038	10
Sangano	3	1.73%	4,180	7
Base Camp	1	0.58%	5,096	2
Kashojwa B	2	1.16%	11,500	2
Kabazana A	1	0.58%	10,061	1
**TOTAL**	**173**	**100.00%**	**47,075**	**37**

#### Person characteristics.

There was a total of 173 cases of cholera, 7 confirmed and 166 suspected cases, no deaths. All case-patients were refugees. The case-patients’ ages ranged from 0.1 to 80 years, Median value of 12 (IQR 5-24). The highest percentage (37%) of the case-patients was in the (0-9) age group. Of the case-patients, 65% (112/173) were female. All case-patients had diarrhoea, vomiting, abdominal pain and dehydration, and 68.2% had generalised body weakness (Table 2).

**Table 2:** Case-patient characteristics.

Variable	Frequency	Percent	Cum. Percent
**Sex**			
Females	112	64.74%	64.74%
Males	61	35.26%	100%
**Age group**			
0-5yrs	45	26.01%	26.01%
6-9yrs	19	10.98%	36.99%
10-19yrs	52	30.06%	67.05%
20-39yrs	31	17.92%	84.97%
40-69yrs	20	11.56%	96.53%
70+	6	3.47%	100.00%
**Symptoms**			
Diarrhoea	173	100.00%	100.00%
Vomiting	173	100.00%	100.00%
Abdominal pain	173	100.00%	100.00%
Dehydration	173	100.00%	100.00%
Generalised body weakness	118	68.2%	68.2%

#### Time characteristics.

**Fig 2:**
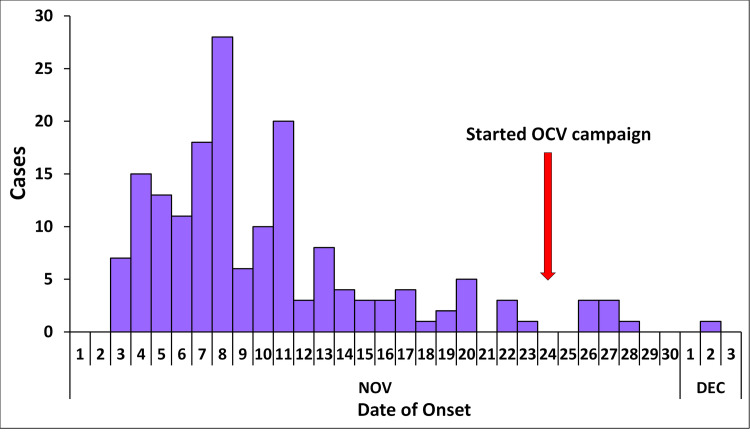
Epidemic curve showing symptom onset dates of persons with suspected and confirmed cholera: Isingiro District, Uganda, November to December 2021.

The date of symptom onset of the first case was 3^rd^ November, 2021. The last case had a date of symptom onset on 2^nd^ December, 2021. The date of discharge of the last confirmed case 5^th^ December, 2021. The number of cases rose rapidly to a peak on 8^th^ November, 2021 and fell off gradually. The OCV campaign was started on 24^th^ November, 2021 (Fig 2).

### Environmental assessment

**Fig 3:**
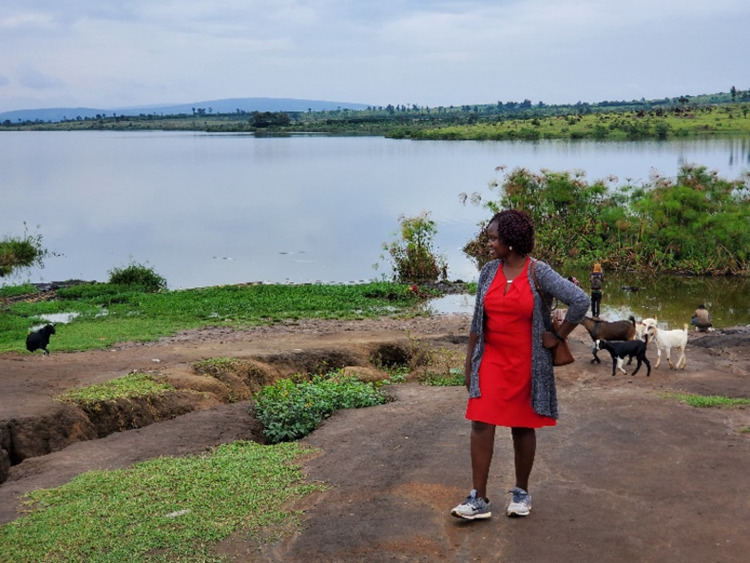
The lake water which is contaminated.

The major water sources in the community are boreholes, taps and lake water. In the affected communities, the biggest problem noted was the lack of consistent safe water supply from the gazetted water points resulting into long queues at the fetching points. In addition, the tap water was brownish in colour with a metallic taste as described by the community. Key informants mentioned that these issues forced people into seeking alternative water sources; namely, the lake which is highly contaminated. The health workers had observed that suspected cholera cases were more in homesteads which used lake water. The visual observation and the activities around the lake showed that the water was not clean. The marshes with very slow-moving water are where most of the water is collected by the community as compared to the clear free-flowing water of the lake (Fig 3). Most households had latrines and were generally clean with a few cases of open defecation observed in the communities. Few homes also had hand washing facilities close to the latrines.

### Water test results

The 2 public boreholes of Nyarugugu tested negative to bacteria contamination. All the 4 samples picked from households tested positive to bacteria contamination irrespective of the sources of water. Samples 7 and 8 which were lake water showed the highest contamination and the colonies couldn’t be counted (Table 3).

**Table 3:** Bacteriological water testing results.

Sample no.	Sample name	Sample description	No. of bacteria colonies per 100ml of sample	National standards for portable water
1	Distilled water	–	0	0
2	Nyarugugu B borehole	Sampled from the borehole	0	0
3	House hold 1 (Nyarugugu B)	Rain water sampled from a Jerrycan	30	0
4	House hold 2 (Nyarugugu B)	Rain water sampled from a household storage tank	86	0
5	Nyarugugu A borehole	Sampled from a borehole	0	0
6	House hold 3 (Nyarugugu C)	Borehole water sampled from a Jerrycan	20	0
7	House hold 4 (Nyarugugu C)	Lake water sampled from a Jerrycan	Numerous	0
8	Lake water	Sampled from a lake	Numerous	0

### Reactive oral cholera vaccination campaign

The vaccine for the Oral Cholera Vaccination campaign (OCV) that has been used since 2018 in Uganda is Euvichol plus (Eubiologics, Korea). The first dose of OCV was done from 21^st^ to 28^th^ November 2021 targeting the three most affected villages of Nyarugugu A, B and C. The total population of these villages was 15,198 persons and the target population (one year and above) was 14,355 persons. For the first campaign 11,356 people were vaccinated and the vaccination coverage was 79.1%. The second dose of the vaccination was done two weeks later and 11,111 people were vaccinated (10,616 got second dose and 495 got first dose). For the second dose campaign, the vaccination coverage was 93.5% (10,616/11,356) for those receiving the second dose.

## Discussion

The cholera outbreak in Nakivale Refugee Settlement in Isingiro district was likely caused by consumption of contaminated lake water. It affected 173 refugees from 3^rd^ November to 2^nd^ December 2021 and the most affected villages were Nyarugugu B and C. The OCV campaigns were conducted in the three villages of Nyarugugu A, B and C with a coverage of 93.5% for two vaccine doses received.

The villages that were affected by the cholera outbreak were all in Kashumba sub county and lie in closest proximity to Lake Nakivale. Kashumba sub county shows the largest population of all sub counties and the greatest percentage is in the settlement [13]. It was observed in the affected villages that although there are designated water points from boreholes and taps, they are insufficient therefore the communities seek alternative water sources from the lake which are usually the marshes with very slow flowing water. There is a possibility of post contamination of water after it has been drawn from the source, probably by using the same container that is used to carry lake water which is highly contaminated. This was observed in samples 3 and 4 where rain water tested positive to bacteria contamination which is as a result of water pollution by human and animal waste.

Water supply is a major issue in the settlement and although there is a large amount of surface water resources in the district, limited infrastructure and absence of water management systems has caused insufficient water for agricultural production and domestic use [13]. Critical issues include the cost implications for water treatment, the increasingly polluted water in Lake Nakivale and the limited waste management system [13].

Refugee settlements have been known to be high risk areas for cholera outbreaks [10]. Also the cholera risk is high among communities living near a lake with the highest incidences occurring with a decrease in distance to the nearest lake which was seen in this outbreak with the most clusters of cases in villages in closest proximity to the lake [15]. The open defecation observed in the refugee communities can be a source of lake water contamination. Cholera transmission is closely associated with insufficient access to clean water and sanitation facilities [5]. A study in the Democratic Republic of Congo found that the increased number of suspected cholera cases, the aggravation of conflict events and the number of internally displaced people (IDPs) in eastern endemic areas were associated with an increased risk of cholera spreading outside the endemic eastern provinces [16]. A cholera outbreak in a refugee camp in Kenya in 2009 revealed that the presence of dirty water storage containers was a risk factor for cholera [17]. In another cholera outbreak in a refugee camp in Kenya between November 2015 and June 2016, the subcamps with poorer water drainage had the highest rates of cholera [18].

The cholera outbreak affected all age groups although the age group (10-19) years had the highest proportion of case-patients at 30.1% followed by the age group (0-5) years at 26%. A cholera epidemic in Nigeria found the age group (5-9) had the highest proportion of cases [19] and an outbreak in the Democratic Republic of Congo showed that the age group (5-14) years was most affected [20]. Both males and females were affected, females had the highest proportion of case-patients affected at 65% similar to findings from a Cholera outbreak in Nigeria with 58.1% females affected [19]. Cholera is mainly transmitted through contaminated water and food [5]. Women and girls have a greater risk of getting into contact with a high infectious dose of cholera due to the domestic roles they traditionally hold in the home and in the community as carers and caregivers [21]. However enhanced surveillance in seven zones and four outbreak sites in Togo, the Democratic Republic of Congo (DRC), Guinea, Uganda, Mozambique and Cote d’Ivoire found that confirmed cholera cases were more likely to be male [22].

The epidemic curve showed a point source pattern of spread where persons could have been exposed to the same common source over a brief period of time. The majority of cases occurred within one incubation period. The OCV campaign was started about two weeks when the outbreak was confirmed and the two doses were given with two weeks delay between each dose. Thereafter there was a decline in number of cholera cases. The OCV campaign was done in conjunction with other control and prevention strategies as recommended including surveillance, water, sanitation and hygiene and social mobilisation [5]. Lessons learned include the importance of having a good surveillance system to quickly pick up suspected cases of cholera and the presence of a cholera treatment centre which enables optimum management of cholera patients.

### Study limitations

This study’s findings may be subject to a potential underestimation bias, as our analysis relied on data from a single health centre’s line list, which might not have captured all cases from the broader community. Unreported cases may have gone undetected, potentially masking the true extent of the outbreak. Active case search revealed that there were some people in the community with cholera-like symptoms however these were few and although they did not go to the health centre for medical treatment we think their number has minimal effects on our findings. Also, no survey was conducted with a standardised data collection tool. Furthermore, lake water testing revealed substantial microbial contamination. However, the absence of specific source attribution analysis precludes establishing a direct causal link between lake water and cholera outbreaks. Our results should be interpreted within the context of this methodological limitation. In addition, the tap water was not collected and analysed which would have been crucial considering that water crisis was being linked to cholera.

## Conclusion

The cholera outbreak at Nakivale Refugee settlement was likely caused by consumption of contaminated lake water and the OCV campaign helped to control the outbreak. There is a need for the Nakivale Refugee settlement to be provided with plentiful supply of water through an effective water management system, treatment of lake water and good infrastructure.

## Supporting information

S1 Data Cholera Outbreak at Nakivale Refugee Settlement, Isingiro District, Uganda. (XLS)
